# Quantification of cephalomedullary nail fit in the femur using 3D computer modelling: a comparison between 1.0 and 1.5m bow designs

**DOI:** 10.1186/s13018-016-0389-7

**Published:** 2016-04-27

**Authors:** Beat Schmutz, Jayani Amarathunga, Stanley Kmiec, Prasad Yarlagadda, Michael Schuetz

**Affiliations:** Institute of Health and Biomedical Innovation, Queensland University of Technology, 60 Musk Avenue, Kelvin Grove, QLD 4059 Australia; DePuy Synthes, 1301 Goshen Parkway, West Chester, PA 19380 USA; School of Chemistry, Physics and Mechanical Engineering, Science and Engineering Faculty, Queensland University of Technology, 2 George Street, Brisbane, QLD 4001 Australia; Trauma Services, Princess Alexandra Hospital, 199 Ipswich Road, Woolloongabba, QLD 4102 Australia

**Keywords:** Intramedullary nail, Nail fit, 3D modelling, Fracture fixation, Femur, Cephalomedullary

## Abstract

**Background:**

The radius of curvature (ROC) misfit of cephalomedullary nails during anterograde nailing can lead to complications such as distal anterior cortical encroachment. This study quantified the anatomical fit of a new nail with 1.0-m ROC (TFN-ADVANCED^™^ Proximal Femoral Nailing System [TFNA]) compared with a nail with 1.5-m ROC (Gamma3 Long Nail R1.5 [Gamma3]).

**Methods:**

We generated 63 three-dimensional models (48 female, 45 right femur) representing the cortical surfaces of the femora (31 Caucasian, 28 Japanese, and 4 Thai). The mean age of the specimens was 77 years (±8.1), and the mean height was 158.5 cm (±9.6). Utilizing a customized software tool, nail fit was determined from the total surface area of nail protrusion from the inner cortex surface and maximum distance of nail protrusion in the axial plane; the position of the distal nail tip within the canal was also determined.

**Results:**

Overall, TFNA had both a significantly smaller mean total surface area of nail protrusion (915.8 vs. 1181.6 mm^2^; *P* < 0.05) and a mean maximum distance of nail protrusion in the axial plane (1.9 vs. 2.1 mm; *P* = 0.007) when compared with Gamma3. The mean total surface area of nail protrusion was significantly smaller with TFNA versus Gamma3 in both the Caucasian (*P* = 0.0009) and Asian (Japanese and Thai) samples (*P* = 0.000002); the mean maximum distance of TFNA protrusion was significantly smaller in Asians (*P* = 0.04), but not in Caucasians (*P* = 0.08). Most tip positions for both nail types were anterior, but TFNA had a higher number of center positions than Gamma3 (13 vs. 7) and a shift from the far anterior cortex to the center of the medullary canal (overall and in Caucasians). In Asians, the most prominent position was far anterior for both nails.

**Conclusions:**

The 1.0-m ROC TFNA nail resulted in better fit than the 1.5-m ROC Gamma3 nail. Clinical trials and case studies should be conducted in the future to verify if these findings would also result in clinical improvements.

## Background

Despite the decrease of nail radius of curvature (ROC) over the last decades, and the generally good results [1], recent clinical studies [2–4] still report the existence of misfit between certain patient anatomy and nail designs during anterograde nailing, which can lead to complications such as distal anterior cortical encroachment.

Anterograde nailing using cephalomedullary fixation is the standard treatment for femoral shaft fractures. Fracture healing with an intramedullary nail inserted into the femur is highly effective, with union rates of 95–99 % reported [1]. However, clinical experience from recent studies has shown that a radius of curvature of 1.5–3.0 m sometimes leads to postoperative complications [2–4]. For example, when using nails with different ROCs during intramedullary nailing for subtrochanteric fractures, it was the difference in femoral anteroposterior bow between the bone and the implant which contributed to distal anterior cortical encroachment [2]. Another study reported a 16 % rate of cortical encroachment with nails with an ROC of 1.8 m [3]. Importantly, better outcomes have been achieved with nails with smaller ROCs. For example, Collinge and Beltram reported rates of abuttal to the distal femur’s anterior cortex of 12 % with a 2.0-m ROC nail, but only 3 % with a 1.5-m ROC nail [4]. Thus, continuing the decrease of nail ROC to more closely match anatomical measurements may help to improve implant fit and potentially help to reduce cortical encroachment.

Gamma nails are intramedullary nails manufactured by Stryker (Kalamazoo, MI, USA). In 2006, over one million Gamma nails had been implanted worldwide [5]. In 2012, Gamma nails were still a trusted and preferred option, representing the highest proportion of US market sales (65 %) [6]. Considering their industry benchmark status, Gamma3 nails were therefore chosen as a viable, robust, and valid comparator for this study.

Computer graphical methods can be used to provide a quantitative fit assessment of implants or fracture fixation [7, 8]. Recently, a comprehensive anatomical modelling study was conducted to establish the anatomical bow of the femur [9]. Three-dimensional (3D) models of the outer and inner surface of the bone cortex were generated from computed tomography (CT) scans using standard protocols, based on samples derived from Caucasian (*n* = 47) and Japanese (*n* = 28) subjects aged 65–103 years [10–12]. The modelling showed a mean ROC of 0.97 m for the Caucasian subjects and 0.78 m for the Japanese subjects [9]. Based on these observations, the TFN-ADVANCED^™^ Proximal Femoral Nailing System (TFNA; DePuy Synthes, West Chester, PA, USA) was designed with a ROC of 1.0 m to more closely match anatomical measurements.

The purpose of the study was to quantify through 3D computer modelling whether the new TFNA nail, with a 1.0-m bow design, provides a better anatomical fit compared with an existing nail with a 1.5-m bow design (Gamma3 Long Nail R1.5 [Gamma3]; Stryker Trauma GmBH, Schönkirchen, Germany).

## Methods

### 3D bone models

For the current study, 63 3D models representing the outer and inner cortex surfaces of the femora were utilized. Of these, 48 were female and 45 were of the right femur. Of the subjects, 31 were of Caucasian origin, 28 of Japanese origin, and 4 of Thai origin. The mean age of the specimens was 77 years (standard deviation [SD] 8.1 years; range 65–103 years) with a mean height of 158.5 cm (SD 9.6 cm; range 143–178 cm). All the bone models were considered to be of normal appearance. The bone models were generated based on specimens from two separate sources: 41 were extracted from the DePuy Synthes bone model database and 22 were generated from CT scans of isolated Caucasian cadaver femora.

CT image data from these specimens were reconstructed into 3D models using commercial software (Amira; FEI, Hillsboro, OR, USA) according to a standard protocol developed by the authors’ research group [12].

### Nail design and fit

TFNA and Gamma3 nails were assessed. The nail length and diameter for each bone model were chosen by clinical conventions, with nail configurations encompassing the following ranges of implants: the nail measured 300–420 mm in length; had a diameter of 10–11 mm; and had a collodiaphyseal angle of 125–130°.

To create digital files of the Gamma3 nail, first, point cloud data were collected using a Surveyor DS2030 (Laser Design Inc., Minneapolis, MN, USA) coordinate measuring machine base, with an RPS-120 (Laser Design Inc.) triangulation non-contact laser probe. Then, the point cloud data were transformed into accurate 3D native computer-aided design models using Geomagic^®^ Studio (3D Systems, Inc., Rock Hill, SC, USA) and comparisons were run using Polyworks/IMView^™^ software (InnovMetric Software, Inc., Québec, QC, Canada). Finally, digital files of the nails were created using the reverse engineering software package Rapidform 2006 (Inus Technology Inc., Seoul, Korea).

### Nail entry point, insertion depth, and axial alignment

**Fig. 1:**
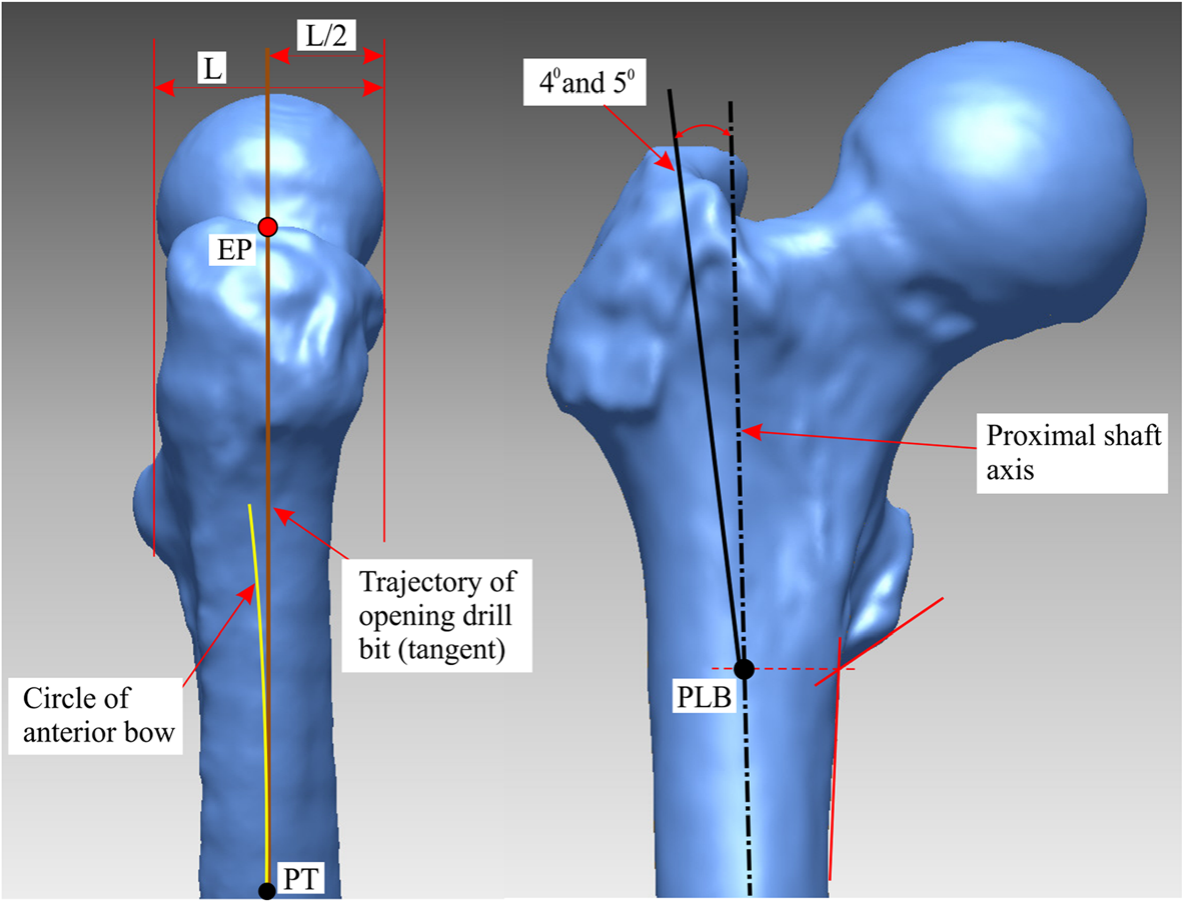
Nail insertion entry points shown for the right femur: lateral (*left*) and anteroposterior (*right*) views. Abbreviations: *EP* nail entry point, *L* lateral, *PT* point of tangency

As the selection of the entry point can have a significant impact on the nail fit, the entry point was clearly defined [13]. For Gamma3 nails, the manufacturers’ operative technique guide was initially used to define the entry point: at the junction of the anterior third and posterior two thirds of the tip of the greater trochanter and on the tip itself [14]. However, based on the 3D modelling, this entry point resulted in penetration of the nail from the anterior outer bone surface in the subtrochanter area. Therefore, the nail entry point was further refined to ensure a fair comparison; the lateral view and anteroposterior views for the nail entry points are shown in Fig. 1. In the anteroposterior view, two different entry points were used to accommodate the different lateral nail bends: lateral from the proximal shaft axis 4° and 5° for Gamma3 nails and TFNA nails, respectively. For nail insertion depth, the nail was inserted until the proximal locking element axis passed through the center of the femoral head. For axial rotational alignment, the nail was rotated until the axis of the proximal locking element was aligned with the center of the femoral head.

### Quantification of nail fit

The inner surface of the bone cortex was used as the criterion to determine anatomical nail fit because this represents the available space for nail positioning. If the nail protruded at the inner surface, then this was classified as a misfit. Once the proximal end of the nail was centered at the entry point, the nail tip position was adjusted to obtain optimal fit. A software tool, previously developed by the authors for tibial nail fit assessment, was modified and used to automate the process of finding the nail position in order to result in the smallest area of protrusion [15]. For all bone models, the nail fit was quantified for the unreamed case because this represents the most challenging situation for achieving anatomical fitting.

### Outcomes

**Fig. 2:**
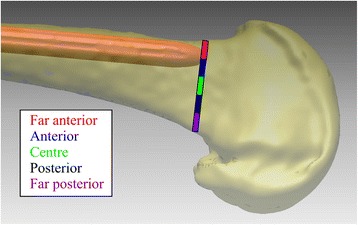
Schematic of final nail positions

The outcomes measured were the total surface area of nail protrusion from the inner cortex surface and the maximum distance of nail protrusion in the axial plane, according to methods previously published [8]. In addition, the position of the distal nail tip within the medullary canal was determined (far anterior, anterior, center, posterior, or far posterior) (Fig. 2). The outcomes were also reported for the Caucasian and Asian (Japanese and Thai) subjects separately. A case study was conducted in which 3D computer modelling was used to assess the nail fit for an average Caucasian sample with an ROC of 1.015 m.

### Statistical analysis

The total surface area of nail protrusion and the maximum distance of nail protrusion generated by the two nail designs were compared using a paired two-tailed Student *t* test calculated using standard statistical software (SPSS Statistics 21.0; IBM, Chicago, IL, USA). A *P* value <0.05 was considered to be a statistically significant difference.

## Results

### Nail protrusion

In the overall sample, the TFNA nail had a significantly smaller mean total surface area of nail protrusion compared with the Gamma3 nail (915.8 vs. 1181.6 mm^2^; *P* < 0.05) (Table 1). The TFNA nail also had a significantly smaller mean maximum distance of nail protrusion in the axial plane compared with the Gamma3 nail (1.9 vs. 2.1 mm; *P* = 0.007) (Table 1).

**Table 1:** Nail protrusion areas and distances

	Overall (*N* = 63)			Caucasian (*n* = 31)			Asian (*n* = 32)		
	TFNA	Gamma3	*P*	TFNA	Gamma3	*P*	TFNA	Gamma3	*P*
Total surface area of nail protrusion (mm^2^)	915.8 (±948.6)	1181.6 (±1157.1)	<0.05	583.3 (±665.6)	792.4 (±914.9)	0.0009	1238.0 (±1073.8)	1558.6 (±1252.8)	0.000002
Maximum distance of nail protrusion (mm)	1.9 (±1.5)	2.1 (±1.8)	0.007	1.3 (±0.9)	1.5 (±1.2)	0.08	2.4 (±1.7)	2.7 (±2.1)	0.04

All values are mean (±SD)

In the 31 Caucasian samples, the mean total surface area of the nail protrusion was significantly smaller with the TFNA compared with the Gamma3 nail (583.3 vs. 792.4 mm^2^; *P* = 0.0009), but the difference in the mean maximum distance of the nail protrusion was not significant (1.3 vs. 1.5 mm; *P* = 0.08) (Table 1).

In the 32 Asian samples, the TFNA nail had both a significantly smaller mean total surface area of nail protrusion (1238.0 vs. 1558.6 mm^2^; *P* = 0.000002) and also a significantly smaller mean maximum distance of nail protrusion (2.4 vs. 2.7 mm; *P* = 0.04) when compared with the Gamma3 nail (Table 1).

### Nail tip position

**Fig. 3:**
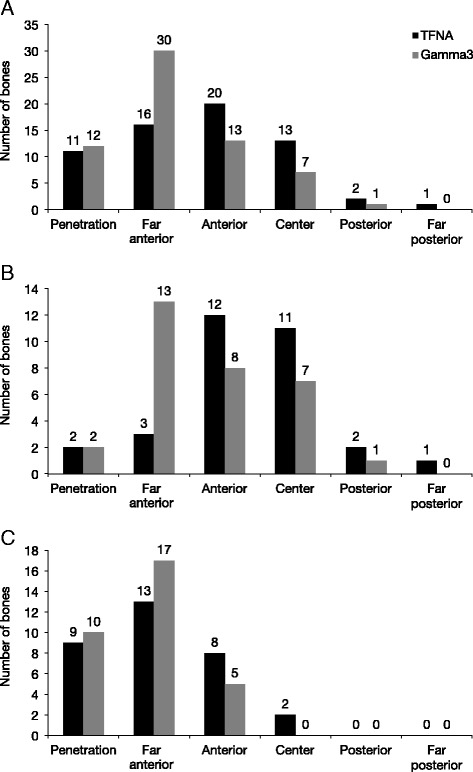
Nail tip positions in five regions of the medullary canal in the overall population (**a**), in Caucasian samples (**b**), and in Asian samples (**c**)

The TFNA nails showed distribution of position across the medullary canal, whereas there were no cases of far posterior positioning for the Gamma3 nail (Fig. 3). The far anterior position for the nail tip within the medullary canal was observed markedly less often with the TFNA nail compared with the Gamma3 nail (16 vs. 30 times) (Fig. 3).

In the Caucasian samples, there was a fourfold reduction in the far anterior positions with TFNA compared with Gamma3 (3 vs. 13) (Fig. 3b). In the Asian samples, there were no center, posterior, or far posterior positions for Gamma3 (Fig. 3c).

**Fig. 4:**
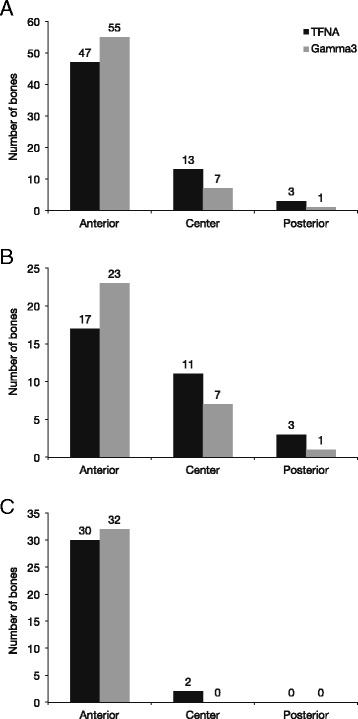
Distribution of nail tip positions relative to the center in the overall population (**a**), in Caucasian samples (**b**), and in Asian samples (**c**)

With regard to position of the distal nail tip relative to the center, the majority of tip positions for both nail types were anterior (Fig. 4). The TFNA nail had a considerably higher number of center positions than the Gamma3 nail (13 vs. 7), and there was a shift of the TFNA nail tip position away from the anterior cortex towards the center of the medullary canal (Fig. 4). This trend was also observed in the Caucasian samples (Fig. 4b); in the Asian samples, the majority of nail positions for both TFNA and Gamma3 were anterior (Fig. 4c).

### Case study

**Fig. 5:**
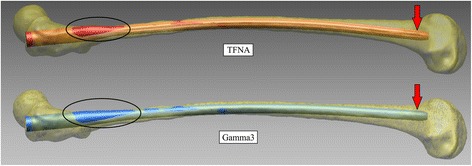
Case study of Caucasian model with an ROC of 1.015 m

Applying 3D computer modelling to an average Caucasian sample with an ROC of 1.015 m resulted in a slightly smaller misfit in the subtrochanteric region for the TFNA nail compared with the Gamma3 nail (Fig. 5). Distally, the TFNA nail achieved a center position whereas the Gamma3 nail showed an anterior position (Fig. 5).

## Discussion

Our 3D computer modelling study found that the TFNA nail had a smaller nail protrusion area and a shorter protrusion distance than the Gamma3 nail. In addition, with the TFNA nail, there was a shift of the distal nail tip away from the anterior cortex towards the center of the medullary canal; this was particularly striking for the far anterior positions which have been reduced by nearly half for the TFNA nail.

Previous studies using 3D modelling to assess quantitative nail fit within the femurs do exist [16–19]. However, these studies were conducted with short nails and with Asian specimens only. Therefore, due to the differences in methodology, implant types, and specimens used, the results of our study cannot be directly compared with prior data. Nevertheless, these previous studies consistently reported mismatches of the nail fit in Asian specimens: with a Gamma nail in both Thai and Chinese subjects; with a retrograde nail in Asian subjects; and with PFNA-II and InterTan in Chinese subjects [16–19].

Previous studies have shown that anatomical misfitting of cephalomedullary nails used for the treatment of femoral shaft fractures may result in distal anterior cortical encroachment or penetration [2, 3]. When we applied our modelling to an average Caucasian sample with an ROC of 1.015 m, this resulted in a slightly smaller misfit in the subtrochanteric region for the TFNA nail compared with the Gamma3 nail. Furthermore, the TFNA nail achieved a center position, whereas the Gamma3 nail showed an anterior position (Fig. 5).

TFNA nail achieved better fit compared with Gamma3 nail overall but also in the subgroups of Asian and Caucasian samples. In Caucasians only, the total surface area of nail protrusion was significantly smaller with TFNA compared with Gamma3, whereas in Asian samples, both the total surface area of nail protrusion and a mean maximum distance of nail protrusion in the axial plane were significantly smaller with TFNA compared with Gamma3. In terms of nail positioning, there seemed to be a trend, attributed to a smaller bow radius, for fewer far anterior positions and more anterior/center positions for TFNA when compared with Gamma3. Nevertheless, the majority of tip positions were still anterior for both nails, particularly in the Asian samples. In a separate study, the mean ROC was 0.78 m for the Japanese subjects and 0.97 m for Caucasians, which might explain why the majority of tip positions were still anterior for both nails, particularly in the Asian samples [9]. These data suggest that smaller stature bones—such as those observed in Asian subjects—may need an even smaller ROC. However, future simulation and clinical studies are required to determine if, and by how much further, the bow radius of a long nail can be further reduced without compromising the safe insertion of the nail.

As with any theoretical modelling study, our analysis has several limitations. Firstly, our study was based on 63 samples. Studies with a higher volume of specimens may have yielded more robust results, particularly for the ethnic groups analyzed. Secondly, the nails were inserted into the intact bone, which is not necessarily representative of clinical cases. In addition, we only measured geometric nail fit and did not account for deformation of the nail or the bone upon nail insertion, phenomena which have been observed in clinical practice. We are currently working on a project aiming to address the latter issue.

## Conclusions

In conclusion, the TFNA nail with a 1.0-m bow design resulted in a better fit (evaluated by protrusion area, protrusion distance, and far anterior nail tip positions) compared with the Gamma3 nail with a 1.5-m bow design. Clinical trials and case studies should be conducted in the future to verify if these findings would also result in clinical improvements.

## Ethics statement

Part of the CT data for our study was available from the industry partner’s database. This database has been populated with de-identified data that was acquired from scans of cadaver specimens in Europe, USA, and Asia according to industry standard best practices. The remaining CT data was derived from de-identified and isolated cadaver femora specimens, which were obtained from an American registered Anatomical Tissue Bank, as part of the industry partner’s implant development activities. According to the NIH Grant and Funding Policy, research that proposes the use of only cadaver specimens is not human subject research under HHS regulations at 45 CFR Part 46, because, by definition, human subjects must be “living individuals.” On this basis, ethics approval was not required.
